# Nationwide Investigation of Respiratory Problemsin Sheep Lambs and Goat Kids in Greece

**DOI:** 10.3390/ani15213155

**Published:** 2025-10-30

**Authors:** Eleni I. Katsarou, Charalambia K. Michael, Dafni T. Lianou, Dimitra V. Liagka, Georgia A. Vaitsi, Vasia S. Mavrogianni, George C. Fthenakis

**Affiliations:** 1 Veterinary Faculty, University of Thessaly, 43100 Karditsa, Greece; elekatsarou@vet.uth.gr (E.I.K.);; 2 School of Veterinary Medicine, European University of Cyprus, Nicosia 2404, Cyprus; 3 Faculty of Animal Science, University of Thessaly, 41110 Larissa, Greece

**Keywords:** kid, lamb, pneumonia, respiratory

## Abstract

**Simple Summary:**

This work investigated respiratory problems in lambs and kids in Greece, in an extensive study performed throughout the country, by means of an in-person questionnaire investigation. The annual incidence rate of respiratory problems in lambs and kids was found to be 1.4% and 1.1%. The proximity (<10 km) of the farm to industrial sites emerged as a significant predictor in sheep and goat farms, which is a notable finding of the study; other significant predictors were found to be the lack of a barn for lambs, the experience of farmers, and the routine administration of antibiotics to newborns. Respiratory problems were considered as important health problems by 26% of farmers. The present results have some similarities with those of relevant studies in people and potentially reflect that air pollution in the farm environment might be a factor to take into account in health management.

**Abstract:**

This study, carried out as part of a large countrywide investigation into the sheep and goat industries in Greece, focused on respiratory problems of lambs and kids in Greece. The work was performed as part of a wider study performed in farms throughout the country with the participation of farmers, by means of an in-person questionnaire investigation. The specific objectives of the study were (a) the assessment of the presence of respiratory problems in lambs and kids and (b) the identification of variables associated with the presence of these problems in the farms. Data were collected from 325 sheep flocks and 119 goat herds. The annual incidence rate for respiratory problems in lambs was 1.4% (95% confidence intervals: 1.3–1.4%) and that in kids was 1.1% (1.0–1.2%). The annual incidence rate was significantly lower in farms that applied a semi-extensive or extensive management system (1.2% in sheep and 1.0% in goat farms) than in farms that applied an intensive or semi-intensive or extensive (1.5% and 1.3%, respectively) management system. In multivariable analysis, the lack of a barn for lambs, the proximity (<10 km) of the farm to industrial sites, and the experience of farmers emerged as significant predictors in sheep farms, and the proximity to industrial sites and the administration of antibiotics to newborns routinely emerged as significant predictors in goat farms. Sheep (27.4%) and goat (22.7%) farmers considered ‘pneumonia’ as the second most important health problem of lambs and kids. Respiratory problems were more often declared an important problem by farmers in proximity to industrial sites: 21.6% versus 12.5%. Overall, the study contributes information regarding the presence of respiratory problems in lambs and kids in Greece. A notable finding has been the association of proximity to industrial sites with a higher incidence rate of respiratory problems of lambs and kids in the farms. This has similarities to the results of relevant studies on people and potentially reflects that air pollution in the farm environment might be a factor to take into account in health management. One may also postulate that, possibly, data from farms can be employed to indicate potential risk from air pollution for humans, although further and more detailed work will be necessary to draw relevant conclusions.

## 1. Introduction

Respiratory infections of lambs and kids have a financial significance in small ruminant farming, because they lead to losses of young animals, as well as to reduced bodyweight gain of affected animals [1,2]. Moreover, treatment of the affected animals requires the increased use of antibiotics in the farm; in a study undertaken in France, it was found that antibiotics administered against respiratory infections of lambs and kids accounted for most of the antimicrobials used in sheep and goat farms [3], which thus contributes to potential emergence of antibiotic resistance in causal bacteria. Management-related variables are considered of particular importance as predisposing factors for respiratory infections of lambs and kids. Among them, weaning [4] and transportation [5] of lambs or kids can precipitate respiratory infections, as they lead to stress of the animals and, thus, reduce immune response ability. Increased concentration of ammonia within animal barns can also play a predisposing role [6]. Further, host-related factors are also important, through the increase in local mucosal irritation [7,8].

Various pathogens are implicated in the etiology of respiratory infections in lambs and kids, which have been cumulatively termed as ‘ovine respiratory complex’ [2]. These include primarily *Mannheimia haemolytica* and *Bibersteinia trehalosi* as the main pathogens responsible for the problem [9,10]. Other microorganisms isolated from cases of infections are *Pasteurella multocida*, *Mycoplasma* spp. (e.g., M. *ovipneumoniae*) and *Escherichia coli*, but these are less frequent etiological agents [11,12]. Finally, occasional recoveries include *Trueperella pyogenes*, *Staphylococcus* spp., *Streptococcus* spp. and *Pseudomonas* spp. [13].

In Greece, the small ruminant industry is the most important sector of animal production, given that its contribution amounts to 1% of the total annual gross domestic product of the country [14]. Previous studies performed in the country focused on respiratory infections in adult sheep caused by Lentivirus [15,16,17], while the extent of respiratory problems in lambs and kids has not been investigated.

This study, carried out as part of a large countrywide investigation into the sheep and goat industries in Greece, focused on respiratory problems of lambs and kids in Greece. The work was performed as part of a wider study performed in farms throughout the country with the participation of farmers, by means of an in-person questionnaire investigation. The specific objectives of the study were (a) the assessment of the presence of respiratory problems in lambs and kids and (b) the identification of variables associated with the presence of the problems in the farms.

## 2. Materials and Methods

### 2.1. Visits to Farms

**Figure 1:**
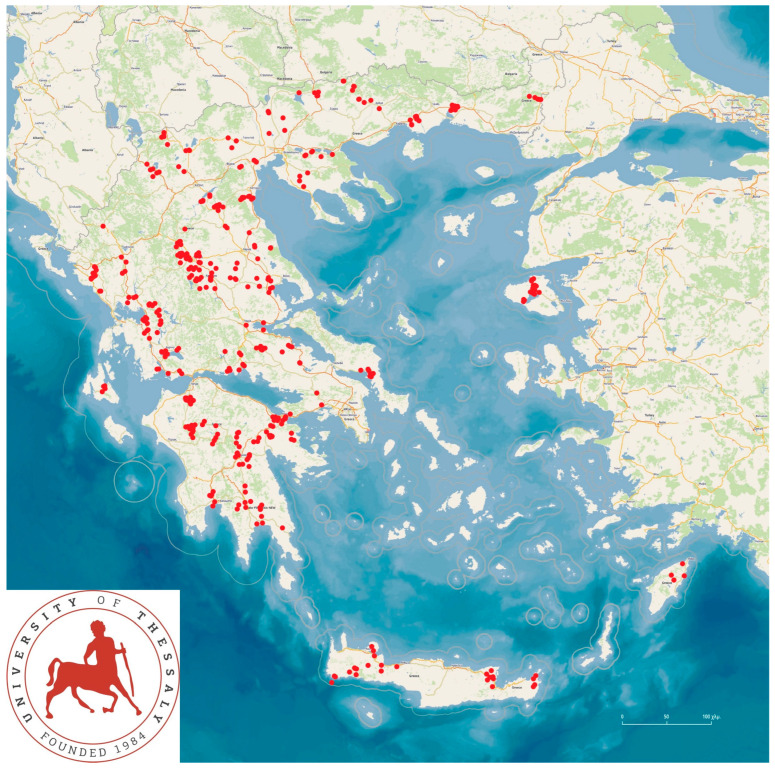
Location of the 444 small ruminant farms (locations indicated by red dots) throughout Greece, which were visited during the investigation [18].

The work was performed as a part of a large countrywide field investigation, with visits made by the investigators to the farms that participated in the study. In total, 325 sheep flocks and 119 goat herds were visited for collection of data and information. All farms were commercial enterprises of the dairy production type, which is the predominant sheep/goat farming mode in Greece. There were in total 110,228 sheep and 30,192 goats on the farms into the study. The mean number of ewes on sheep farms was 325 (standard error (s.e.): 13) and the mean number of does on goat farms was 237 (s.e.: 20). The farms were located in all 13 administrative regions of the country (Figure 1).

Among sheep flocks, 44 (13.5%) were managed under the intensive, 140 (43.1%) under the semi-intensive, 116 (35.7%) under the semi-extensive, and 25 (7.7%) under the extensive management system. Among goat herds, 9 (7.6%) were managed under the intensive, 29 (24.4%) under the semi-intensive, 61 (51.3%) under the semi-extensive, and 20 (16.8%) under the extensive management system (the management system was determined according to the system of the European Food Safety Authority [19]). The annual milk yield obtained per female animal during the preceding milking season was 207 (s.e.: 5) L per ewe and 201 (s.e.: 10) L per doe. In Greece, the terms ‘lambs’/‘kids’ refer to the young animals in small ruminant farms up to the age of slaughter, traditionally 60 to 65 days for lambs and 75 to 90 days for kids. In farms in the study, the average age of slaughter was 50 (s.e.: 1) days for lambs and 65 (s.e.: 3) days for kids. The mean total number of lambs/kids born per female animal during the preceding lambing/kidding season was 1.33 (s.e.: 0.1) lambs and 1.30 (s.e.: 0.2) kids [20].

The inclusion of farms into the study was made on a convenience basis, depending on the willingness of the farmers to be visited by university personnel for an interview [21]. All farms were visited by the researchers, and an interview of the farmer was carried out by using a structured detailed questionnaire, tested before the start of the study for content validity [21]. In order to maintain consistency in the interviewing procedure, all the interviews were carried out by the same person (author D.T.L.). Finally, information obtained from the farmers was verified during a physical evaluation of the farm site and from the veterinarians supervising the farms [21].

### 2.2. Data Management and Analysis

During the visit to each farm, data on farm location were collected using hand-held Global Positioning System Garmin units. The geo-references were resolved to the specific farm level.

The data were entered into Microsoft Excel and analyzed using SPSS v. 21 (IBM Analytics, Armonk, NY, USA). Basic descriptive analyses were initially performed, and exact binomial confidence intervals (CIs) were obtained.

The outcome considered was ‘annual incidence rate of respiratory problems of lambs/kids’. In total, 41 parameters (Table S1) were evaluated for potential association with each of these outcomes. The general categories of these variables were (a) variables related to infrastructure in farms, (b) variables related to animals on the farm, (c) variables related to production characteristics in farms, (d) variables related to health management in farms, (e) variables related to human resources in farms, (f) variables related to climatic conditions at the locations of farms. Climatic variables used in the analysis were derived from ‘The POWER (Prediction of Worldwide Energy Resources) Project’ (NASA Langley Research Center (LaRC), Hampton, VA, USA), which provides meteorological datasets from NASA research for the support of agricultural needs [22].

Standard methodologies for univariable and multivariable analyses were followed, as described in detail by Katsarou et al. [23]. Variables found with *p* < 0.20 in the univariable analyses were offered to the multivariable model [24]. Thus, 18/10 variables were offered to the model for sheep/goat farms, respectively. The variables included in the multivariable models constructed are detailed in Table S2. Subsequently, associations between the parameters into the final multivariable assessment were evaluated by principal component analysis.

Finally, a multivariable analysis model was constructed and assessed, wherein only the management system applied in farms and the proximity (<10 km) of the farm to industrial sites were offered. Subsequently, principal component analyses were carried out between the parameters into the final multivariable assessment for two cohorts of farms separately; one cohort included farms with proximity to industrial sites, and one cohort included farms with no proximity to industrial sites. The Eigenvalues calculated in the principal component analysis for each of the two cohorts were compared between them by Pearson correlation analysis.

Given that in this study, a wide dataset was employed for the identification of potential predictors, prefiltering of variables for inclusion in the multivariable model was repeated by using the value *p* < 0.10, in accordance with the methodology of Hyde et al. [25]. This resulted in a smaller number of variables offered into the multivariable models, as well as a smaller number of variables included in the final model (Table S2).

Separate analyses were performed for sheep flocks and goat herds. In all analyses, statistical significance was defined at *p* < 0.05.

## 3. Results

### 3.1. Descriptive Findings

Among sheep farms, the incidence rate varied from 0.0% to 21.4%; the median value was 0.0% per farm (interquartile range: 0.8%) and the mean value 1.3% per farm (standard error (s.e.): 0.2%). Among goat farms, the incidence rate varied from 0.0% to 26.7%; the median value was 0.0% per farm (interquartile range: 0.0%) and the mean value was1.0% per farm (s.e.: 0.3%) (*p* = 0.052 between sheep and goat farms). Table 1 shows the incidence rate of respiratory problems in accordance with the management system applied on the farms.

### 3.2. Predictors

**Figure 2:**
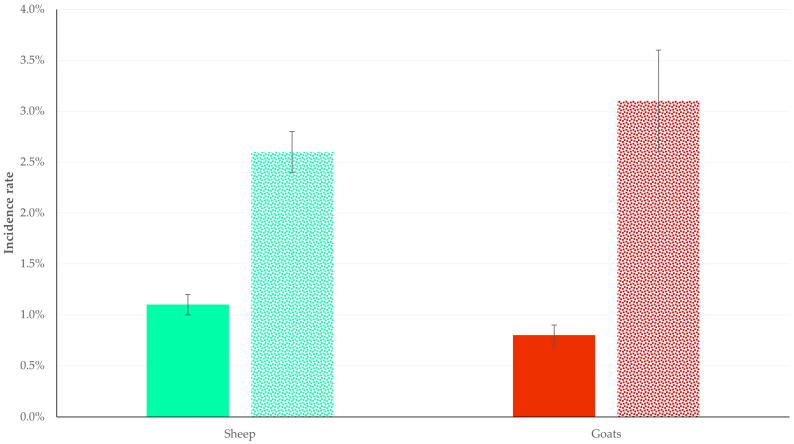
Bar plot for the incidence rate (95% confidence intervals) of lamb/kid respiratory problems in sheep flocks (green bars) and goat herds (red bars), in accordance with the proximity of the farms to industrial sites (full/motif pattern: no proximity/proximity to industrial sites).

The results of the univariable analyses are in Tables S3 and S4. In the multivariable analysis for respiratory problems in lambs, (a) the lack of a separate barn for lambs (*p* = 0.0001/*p* < 0.0001) and (b) the proximity (<10 km) of the farm to industrial sites (*p* = 0.032/*p* = 0.042) emerged as the significant predictors (Table 2 and Figure 2) with the use of either prefiltering values (*p* < 0.20/*p* < 0.10). In the multivariable analysis for respiratory problems in kids, (a) the proximity (<10 km) of the farm to industrial sites (*p* = 0.0004/*p* < 0.0001) and (b) the routine administration of antibiotics to newborns (*p* = 0.011/*p* = 0.015) emerged as the significant predictors (Table 2 and Figure 2) with the use of either prefiltering values (*p* < 0.20/*p* < 0.10).

**Figure 3:**
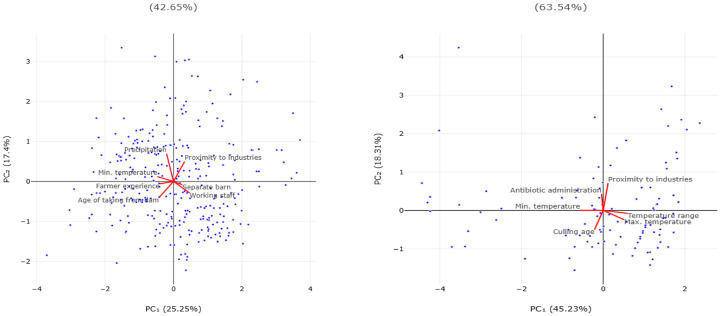
Bi-plot of results of principal component analysis for the parameters in the final multivariable assessments for incidence rate of lamb or kid respiratory problems in sheep flocks (**left plot**) and goat herds (**right plot**) (vectors clockwise from top right: minimum temperature at 2 m for the year preceding the visit, precipitation for the year preceding the visit, proximity (<10 km) to industrial sites, availability of a dedicated building for lambs, presence of working staff at the farm, age for lamb removal from their dams, length of previous animal farming experience (**left plot**), minimum temperature at 2 m for the year preceding the visit, routine prophylactic administration of antimicrobials to newborns, proximity (<10 km) to industrial sites, maximum temperature at 2 m for the year preceding the visit, temperature range at 2 m for the year preceding the visit, average age of culling does (**right plot**)) (standard scaling, with no rotation during preprocessing).

The principal component analysis for the parameters into the final multivariable assessments (with the use of *p* < 0.20 as the prefiltering values for offering variables into the multivariable models) indicated that the two principal components accounted for 42.7% of the variations in sheep flocks and for 63.5% of the variations in goat herds (Figure 3 and Table 3).

Farms near industrial sites were managed more often under the intensive or semi-intensive management system for both sheep flocks (64.8% versus 55.0%; *p* < 0.0001) and goat herds (75% versus 27.1%; *p* = 0.0007). However, in the multivariable analysis, only the proximity to industrial sites emerged with a significant association for both sheep (*p* = 0.018) and goat *p* < 0.0001) farms, whilst the management system was not found to have a a significant association with the annual incidence rate of respiratory problems, also for both sheep (*p* = 0.018) and goat (*p* < 0.0001) farms (Table S5).

**Figure 4:**
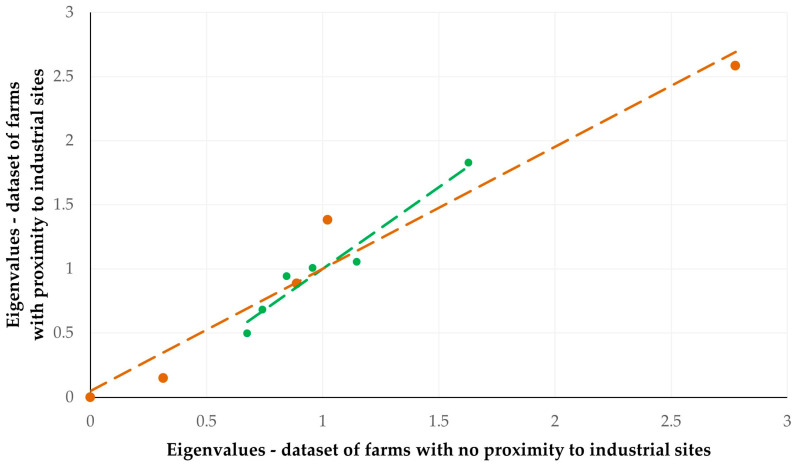
Cross-plot of Eigenvalues calculated in the principal component analysis for each of the two cohorts (coloured dots) (i.e., cohort of farms with proximity to industrial sites and cohort of farms with no proximity to industrial sites) for sheep (green) and goat (red) farms (dashed lines are trendlines).

The comparison of the Eigenvalues calculated in the principal component analysis for each of the two cohorts (i.e., farms with proximity to industrial sites and farms with no proximity to industrial sites) revealed a significant correlation for both sheep (*r* = 0.976, *p* = 0.009 (Figure S1) and goat (*r* = 0.979, *p* = 0.001 (Figure S2)) farms (Figure 4).

### 3.3. Importance for Farmers

Sheep and goat farmers considered ‘pneumonia’ as the second most important health problem for lambs and kids. Specifically, 27.4% of sheep farmers and 22.7% of goat farmers (*p* = 0.32) considered that to be one of the two most important health problems of the animals, in both cases second to diarrhea (Table 4).

There was a clear difference in the annual incidence rate of respiratory problems between farms in which the farmer did or did not declare ‘pneumonia’ as an important pathological condition: 3.7% (3.5–3.9%) versus 0.4% (0.4–0.5%), respectively (*p* < 0.0001). Furthermore, ‘pneumonia’ was declared as an important problem more often by shepherds and goatherds in proximity to industrial sites: 21.6% versus 12.5% (*p* = 0.019).

### 3.4. Administration of Pharmaceuticals and Vaccines to Newborns

The results of association of the annual incidence rate of lamb / kid respiratory problems with the routine administration of antibiotics to newborns in the farms are in Table 5.

Vaccination against bacterial respiratory infections was performed in 144 sheep flocks (44.3% (95% CI: 39.0–49.8%) and 39 goat herds (32.8% (95% CI: 25.0–41.6%) (*p* = 0.028), with a tendency of more frequent application in farms with respiratory problems: 48.7% versus 38.5% (*p* = 0.05).

Farmers who declared ‘pneumonia’ as an important health problem of newborns routinely administered antibiotics to newborns significantly more often than those who did not declare that: 29.3% versus 20.1% of respective farmers (*p* = 0.042). However, no such difference was found for vaccination against bacterial respiratory problems: 45.7% versus 39.6% of respective farmers (*p* = 0.25).

Finally, neither routine administration of antibiotics nor vaccination were found to be performed more frequently in farms with a proximity to industrial sites: in 22.7% versus 22.5% (*p* = 0.97) and in 42.4% versus 41.0% (*p* = 0.83) of respective farms.

## 4. Discussion

### 4.1. Preamble

The current study explored the occurrence of respiratory problems in lambs and kids and factors that may play a role therein, as part of a large field work performed in Greece. Despite the importance of the problem for small ruminant farms and the significance of sheep and goat farming for the primary sector of Greece, the extent of this disorder had not been investigated before. The study involved the participation of farmers, as an in-person questionnaire investigation. The collection of data by means of in-person questionnaire investigation supports ‘*the harvesting of epidemiological intelligence contained within community observations, existing veterinary knowledge and traditional oral history*’ [26]. Advantages of this approach include, among others, the possibility for collection of large amounts of data and flexibility, as well as the transfer of knowledge from rural people regarding issues faced by them [27,28]. By using this approach, it was possible to obtain information from shepherds and goatherds across the country.

The smaller incidence rate of respiratory problems reported in goat herds compared to that in sheep flocks may first reflect differences in susceptibility to various pathogens between the two animal species. For example, incidence of infection by *M. haemolytica* has been found to be higher in sheep flocks than in goat herds [29,30]; furthermore, *B. trehalosi*, which causes disease with severe clinical signs, and is thus easily detectable, was found significantly more frequently in sheep flocks than in goat herds [31]. Second, the differences in the management system applied between the two animal species might have also contributed; goats are more frequently managed under semi-extensive or extensive management systems (68.1% of farms), which reduces the possibility for close observation of kids and detection of clinical signs, whilst sheep are more frequently managed under intensive or semi-intensive management systems (56.6% of farms), which facilitates animal monitoring for clinical signs.

### 4.2. Predictors

The interesting finding of this study was the emergence of the proximity of farms to industrial sites as a predictor for respiratory problems in lambs and kids. Whilst in people, such an association has been well-established [32,33,34,35], the present findings present similar associations in lambs and kids for the first time. Inhalation of air pollutants produced in industrial sites (e.g., fine dust, volatile organic compounds) has been incriminated as a factor predisposing admission to hospital consequent of acute respiratory distress syndrome [35,36], particularly in pediatric patients [37,38]. In other studies, air pollutants have been reported to lead to increased incidence of lung cancer in people living in residential areas near industrial sites [39,40]. Moreover, sources of non-anthropogenic air pollution (e.g., sand dust pollution) can also contribute to downgrading of air quality [41].

Based on these findings, one may postulate a similarity in patterns of the potential effects of industrial pollution to those of people. Notably, the overall average annual particulate matter concentrations in Greece have varied from 17.8 to 20.8 μg m^−3^ in recent years [42], which is higher than the relevant WHO recommendations [43], although significant differences have been identified among locations in the country [44]. Notably, also in Greece, industrial pollution accounts for approximately 30% to 40% of the total air pollution measured in the country [4].

Farms near industrial sites were managed significantly more frequently under the intensive or semi-intensive management system. In such management systems, sheep and goats are housed for long periods [19], in buildings often densely stocked, which can facilitate transmission of pathogens between animals and the subsequent development of respiratory infections [45].

The results of the correlation evaluation of the Eigenvalues obtained during the principal component analysis among cohorts of farms with or without proximity to industrial farms, did not reveal differences between the various components of the analysis between the two cohorts. However, ‘management system’ encompasses a variety of factors, which, to varying extents for each one, can contribute to the development of respiratory problems. In any case, respiratory problems in small ruminants are considered to be of multifactorial nature, which indicates that control should rely on many approaches [2].

In farms, where there was no separate barn for lambs, the newborns were housed in the same buildings as the adult sheep; the consequence of that is the high stocking rate in these buildings. That way, first, there is increased risk for the transmission of respiratory pathogens between the housed animals; furthermore, higher ammonia concentrations will develop within the animal houses. It is well-established that ammonia is a respiratory irritant; furthermore, increased concentrations of ammonia contribute to the deterrence and impediment of the local defenses of the respiratory system of animals, which result from hyperplasia of the nasal mucosa and dysfunction of the cilia [46]. The combination of these two factors contributes to increased incidence of respiratory infections in such cases.

Farmers often attempt to prevent ‘pneumonia’ through the administration of antibiotics; this is an incorrect practice, because it contributes to the development of resistance among bacteria on the farms [47]; hence, it must be discouraged. Appropriate and targeted health management practices should be prioritized in order to effectively control the respiratory problems on a farm, rather than routine prophylactic administration of antibiotics, which may be ineffective, and at the same time also contributes to the development of antibiotic resistance [47].

### 4.3. Importance for Farmers

The two major health problems in young lambs and kids are gastrointestinal and respiratory infections [45], and this has been reflected in the overall answers of the farmers. Nevertheless, the understanding of the significance of the problem did not lead all farmers to apply animal vaccinations against the problem, a possible reason for this being the cost of vaccination. In support of this, it is noted that vaccinations also were not applied more frequently in farms, where predictors for increased incidence of the problem were present.

It can be postulated that farmers prioritize milk production, which is the primary source of income in dairy small ruminant farms. Whilst they recognize the importance of respiratory infections in lambs/kids, they might not be convinced of the financial advantages offered by vaccinations, also given the fact that prices for lamb/kid meat remain low in general, as the result of regular imports from neighboring countries (Bulgaria, Romania), which contribute to continuous supply and lower prices to consumers in the country.

### 4.4. Limitations

The inclusion of farms based on the willingness of farmers to participate (rather than through a randomized inclusion) might have introduced a bias in the study. Nevertheless, even under these circumstances when farms had been included on a convenience basis, refusals of farmers to accept the visit after this had been arranged were recorded [21]. Thus, one may postulate that, in cases of attempting to visit randomly selected farms of unknown farmers, they would be suspicious, unfriendly, and perhaps even hostile, resulting in a higher proportion of refusals.

It may be suggested that through the approach employed in the study, only severe cases of respiratory problems would have been detected and reported by farmers; in contrast, animals with lighter clinical signs would not have been noticed and reported. Nevertheless, Green et al. [48] concluded that even the clinical examination of lambs was an ‘*inefficient and possibly ineffective method to identify lambs*’ with respiratory problems. Therefore, even the clinical examination might not provide accurate identification of cases. Moreover, a clinical examination could only provide a time-spot ‘picture’ of the situation on farms. In contrast, the use of information from farmers, which was verified by veterinarians during the physical visit to the farms, provided extended details about farms throughout the entire year that preceded the visit.

This may possibly be a reason why annual incidence rate of the clinical problems was found to be, in general, smaller than that reported elsewhere. For example, abattoirs studies in Spain and New Zealand indicated that lung lesions in lambs could be present in over 25% of animals evaluated [49,50]. However, in the New Zealand study, severe lesions (which can be comparable to our findings) were found in only 7% of animals [49]; the difference from our findings can potentially be explained to be due to the significantly younger age that lambs/kids in Greece were slaughtered at (50 (s.e.: 1) days for lambs and 65 (s.e.: 3) days for kids [20]), in contrast to New Zealand, where lambs are commonly slaughtered at the age of 8 to 10–12 months, with recommendations for slaughter even up to the age of 14 months [51]. Therefore, the particularly longer fattening period of lambs in New Zealand, compared to lambs in Greece, would have accounted for the large discrepancy in the incidence of respiratory problems between the present findings and those of other works.

Finally, the inclusion of a large number of farms from all parts of the country ensured that conditions practiced all over the country were considered, which resulted in factors of regional importance weighing less. Moreover, environmental conditions prevailing in various parts and regions of the country were also taken into account.

## 5. Conclusions

The study provides data and information about respiratory problems in lambs and kids in Greece. A notable finding is the association of the proximity to industrial sites with a higher incidence rate of respiratory problems of lambs and kids in the farms. This has similarities to the results of relevant studies in people and potentially reflects that air pollution in the farm environment might be a factor to take into account in health management. One may also postulate that, data from farms can possibly be employed to indicate potential risk from air pollution for humans, although further and more detailed work will be necessary to draw relevant conclusions.

## Figures and Tables

**Table 1:** Annual incidence rate of lamb/kid respiratory problems in 325 sheep flocks and 119 goat herds in Greece, in accordance with the management system ^1^ applied in the farms.

	Management System Applied in Farms		*p* Value
	Intensive or Semi-Intensive (*n* = 222)	Semi-Extensive or Extensive (*n* = 222)	
Sheep farms (*n* = 325)	median 0.0% (IQR: 0.8% ^2^) mean 1.5% (s.e.: 0.3% ^3^) range: 0.0–21.4%	median 0.0% (IQR: 0.6%) mean 1.1% (s.e.: 0.2%) range: 0.0–20.0%	0.62
Goat farms (*n* = 119)	median 0.0% (IQR: 0.0%) mean 1.6% (s.e.: 0.8%) range: 0.0–26.7%	median 0.0% (IQR: 0.0%) mean 0.8% (s.e.: 0.2%) range: 0.0–12.5%	0.63
*p* value	0.36	0.12	

^1^ Management system determined according to the system of the European Food Safety Authority [19]; ^2^ IQR: interquartile range; ^3^ s.e.: standard error.

**Table 2:** Results of multivariable analysis for predictors for incidence rate of lamb or kid respiratory problems in sheep flocks and goat herds.

Variables	Relative Risk (±s.e. ^1^)	*p*
Sheep Farms		
Availability of separate barn for lambs		
Yes (0.0% (0.5%) ^2^)	reference	-
No (0.0% (2.4%))	1.014 ± 1.004	0.0008
Proximity of the farm to industrial sites		
Yes (0.0% (2.2%))	1.013 ± 1.005	0.008
No (0.0% (0.5%))	reference	-
Goat Farms		
Proximity of the farm to industrial sites		
Yes (0.3% (6.7%))	1.040 ± 1.009	<0.0001
No (0.0% (0.0%))	reference	-
Routine administration of antibiotics to kids		
Yes (0.0% (3.6%))	1.017 ± 1.009	0.010
No (0.0% (0.0%))	reference	-

^1^ s.e.: standard error; ^2^ median (interquartile range) annual reported incidence rate of respiratory problems among respective farms.

**Table 3:** Eigenvalues for principal component analysis for the parameters in the final multivariable assessments for incidence rate of lamb or kid respiratory problems in sheep flocks and goat herds.

	Sheep Flocks											
Parameter	PC1 ^1^	PC2		PC3		PC4		PC5		PC6		PC7
Eigenvalue	1.767	1.218		0.985		0.832		0.787		0.732		0.678
% of variance	25.25	17.41		14.08		11.88		11.25		10.46		9.69
Cumulative variance (%)	25.25	42.65		56.73		68.62		79.85		90.31		100.0
	**Goat Herds**											
Parameter	PC1		PC2		PC3		PC4		PC5		PC6	
Eigenvalue	2.714		1.098		0.988		0.863		0.337		0.000	
% of variance	45.23		18.31		16.47		14.38		5.63		0.000	
Cumulative variance (%)	45.23		63.54		80.00		94.38		100		100	

^1^ PC: principal component.

**Table 4:** Frequency of the health problems declared by farmers to be the two most important ones among lambs and kids in 325 sheep flocks and 119 goat herds in Greece.

Health Problems ^1^	Sheep Flocks (*n* = 325)	Goat Herds (*n* = 119)
diarrhea	233 (71.7%)	83 (69.7%)
Pneumonia	89 (27.4%)	27 (22.7%)
Contagious ecthyma	25 (7.7%)	4 (3.4%)
Clostridial infections	24 (7.4%)	12 (10.1%)
Coliform infections	12 (3.7%)	11 (9.2%)
Selenium deficiency	10 (3.1%)	<1%
Endoparasitic infections	8 (2.5%)	11 (9.2%)

^1^ As declared by farmers.

**Table 5:** Annual incidence rate of lamb/kid respiratory problems among 325 sheep flocks and 119 goat herds in Greece, in accordance with the routine administration of antibiotics to newborns in the farms.

	Routine Administration of Antibiotics Performed	No Routine Administration of Antibiotics Performed	*p* Value
Sheep farms	0.0% (2.6%) ^1^	0.0% (0.0%)	0.0001
Goat farms	0.0% (3.6%)	0.0% (0.0%)	0.014

^1^ Median (interquartile range).

## Data Availability

Most data presented in this study are in the Supplementary Materials. The remaining data are available upon request from the corresponding authors.

## Supplementary Materials

The following supporting information can be downloaded at https://www.mdpi.com/article/10.3390/ani15213155/s1. Table S1. Information (related to infrastructure, animals, production characteristics, health management, human resources and climatic conditions) obtained for the study of respiratory problems in lambs / kids in 325 sheep flocks and 119 goat herds in Greece; Table S2. Details of multivariable models employed for the evaluation of associations with the incidence rate of lamb / kid respiratory problems in 325 sheep flocks and 119 goat herds in Greece; Table S3. Results of univariable analysis for predictors of incidence rate of respiratory problems in lambs in 325 sheep flocks in Greece; Table S4. Results of univariable analysis for predictors of incidence rate of respiratory problems in kids in 119 goat herds in Greece; Table S5. Complete results of analysis of incidence rate of respiratory problems in lambs and kids in 444 farms in Greece, in accord only with management system and proximity to industrial sites; Figure S1. Bi plot of results of principal component analysis for the parameters into the final multivariable assessments for incidence rate of lamb respiratory problems in sheep flocks with or without proximity to industrial sites (standard scaling, with no rotation during preprocessing); Figure S2. Bi plot of results of principal component analysis for the parameters into the final multivariable assessments for incidence rate of kid respiratory problems in goat herds with or without proximity to industrial sites (standard scaling, with no rotation during preprocessing).
