# Supplementary material for: Nationwide Investigation of Respiratory Problemsin Sheep Lambs and Goat Kids in Greece
Source: Animals (Basel). 2025 Oct 30;15(21):3155. doi: 10.3390/ani15213155 (PMC12610053; doi:10.3390/ani15213155)

Investigation of Respiratory Problems in Sheep Lambs and Goat Kids in Greece through In-Person Questionnaire Approach

Eleni I. Katsarou, Charalambia K. Michael, Dafni T. Lianou, Dimitra V. Liagka, Georgia A. Vaitsi, Vasia S. Mavrogianni and George C. Fthenakis

Table S1. Information (related to infrastructure, animals, production characteristics, health management, human resources and climatic conditions) obtained for the study of respiratory problems in lambs / kids in 325 sheep flocks and 119 goat herds in Greece.

Variables related to infrastructure in farms
Management system applied in farm (EFSA classification: shepherding / intensive / semi-intensive / semi-extensive / extensive / very extensive / mixed)
Part of the country where farm was located (North, Central, South, Islands) Altitude at the location of farm (m)
Availability of a milking parlour (yes / no)
Availability of a dedicated building for lambs / kids (yes / no)
Availability of a separate lambing / kidding area (yes / no)
Proximity (< 10 km) to industrial sites (yes / no)
Variables related to animals on the farm
Number of adult sheep / goats on the farm (no.)
Breed of animals (description)
Variables related to production characteristics in farms
Month of the start of the lambing / kidding season (description)
Average number of lambs / kids born per ewe / doe during the preceding lambing / kidding season (no.)
Average age of culling ewes / does (years)
Variables related to health management in farms
Collaboration with a veterinarian (yes / no) [1]
Total visits made annually by veterinarians to the farm during the preceding season (no.)
Vaccination against bacterial respiratory infections applied in the farm (yes / no)
Age for lamb / kid removal from their dams (days)
Annual frequency of systemic disinfections in the farm (no. of occasions)
Administration of selenium to pregnant animals (yes / no)

Administration of selenium to newborn animals (yes / no)
Routine prophylactic administration of antimicrobials to newborns (yes / no)
Administration of milk replacer to lambs / kids (yes / no)
Application of reproductive control practices in the farm (yes / no)
Source of replacement animals (own animals / purchase)
Newborn care and specific monitoring (yes / no)
Maintenance of a colostrum bank (yes / no)
Lamb / kid fostering to female animals other than their dams (yes / no)
Variables related to human resources in farms
Age of farmer (years)
Length of previous animal farming experience (years)
Highest general education level achieved (primary / secondary / tertiary)
Farmer by profession (yes / no)
Daily period of presence at the farm (hours)
Family tradition in farming (yes / no)
Presence of working staff at the farm (yes / no)
Variables related to climatic conditions at the locations of farms
Temperature at 2 m for the year preceding the visit (^{o}C)
Temperature of Earth skin for the year preceding the visit (^{o}C)
Minimum temperature at 2 m for the year preceding the visit (^{o}C)
Maximum temperature at 2 m for the year preceding the visit (^{o}C)
Temperature range at 2 m for the year preceding the visit (^{o}C)
Relative humidity at 2 m for the year preceding the visit (%)
Precipitation for the year preceding the visit (kg m^{-2} s^{-1})
Wind speed at 10 m for the year preceding the visit (m s^{-1})

[1] In the present context, 'collaboration with a veterinarian' referred to a stable, non-contractual, association with a veterinarian, who, in full accord with and by applying all the relevant professional veterinary conduct codes [Federation of Veterinarians in Europe 2009, Hellenic Veterinary Association 2011], was providing veterinary advice and clinical services in relation to the health and welfare of the animals in the farms [Lianou and Fthenakis 2023].

-Federation of Veterinarians of Europe. *Veterinary Act-European Veterinary Code of Conduct*; Federation of Veterinarians in Europe: Brussels, Belgium, 2009.
-Hellenic Veterinary Association. *Code of Correct Veterinary Practice*; Hellenic Veterinary Association: Athens, Greece, 2011.
-Lianou, D.T.; Fthenakis, G.C. Evaluation of the role of veterinarians for outcomes related to the health and production of dairy small ruminants in Greece. *Animals* **2023**, *13*, 3371.

Table S2. Details of multivariable models ($n = 2$) employed for the evaluation of associations with the incidence rate of lamb / kid respiratory problems in 325 sheep flocks and 119 goat herds in Greece.

Outcome	Variables		
	**assessed in univariable analyses (*n*)**	**offered to the multi-variable models (*n*)**	**required in the final models**
Use of $p < 0.20$ as filter value			
Incidence rate of respiratory problems in lambs	41	18	(a) Availability of a separate barn for lambs, (b) Proximity to industrial sites, (c) Age for lamb removal from their dams, (d) Length of previous animal farming experience of farmer, (e) Presence of working staff at the farm, (f) Minimum temperature at 2 m for the year preceding the visit, (g) Precipitation for the year preceding the visit
Incidence rate of respiratory problems in kids	41	10	(a) Proximity to industrial sites, (b) Average age of culling does, (c) Routine prophylactic administration of antimicrobials to newborns, (d) Minimum temperature at 2 m for the year preceding the visit, (e) Maximum temperature at 2 m for the year preceding the visit, (f) Temperature range at 2 m for the year preceding the visit
Use of $p < 0.10$ as filter value			
Incidence rate of respiratory problems in lambs	41	14	(a) Availability of a separate barn for lambs, (b) Proximity to industrial sites, (c) Administration of milk replacer to lambs, (d) Length of previous a-nimal farming experience of farmer, (e) Presence of working staff at the farm, (f) Precipitation for the year preceding the visit

Incidence rate of respiratory problems in kids	41	6	(a) Altitude at the location of farm, (b) Proximity to industrial sites, (c) Routine prophylactic administration of antimicrobials to newborns, (d) Maximum tem-perature at 2 m for the year preceding the visit

Table S3. Results of univariable analysis for predictors of incidence rate of respiratory problems in lambs in 325 sheep flocks in Greece.

Variable	r_{sp}	p
Management system applied in farm	–0.045	0.41
Part of the country where farm was located	0.127	0.022
Altitude at the location of farm	0.015	0.78
Availability of a milking parlour	0.090	0.10
Availability of a separate barn for lambs	–0.141	0.011
Availability of a separate lambing area	–0.003	0.95
Proximity (< 10 km) to industrial sites	0.169	0.002
Number of adult sheep on the farm	0.070	0.21
Breed of animals	–0.002	0.97
Month of the start of the lambing season	–0.062	0.26
Average number of lambs born per ewe during the preceding lambing season	–0.065	0.24
Average age of culling ewes	–0.059	0.29
Collaboration with a veterinarian	–0.039	0.49
Total visits made annually by veterinarians to the farm during the preceding season	0.036	0.52
Vaccination against bacterial respiratory infections applied in the farm	0.102	0.07
Age for lamb removal from their dams	–0.073	0.19
Annual frequency of systemic disinfections in the farm	0.010	0.85
Administration of selenium to pregnant animals	0.016	0.77
Administration of selenium to newborn animals	0.046	0.41
Routine prophylactic administration of antimicrobials to newborns	0.196	0.0004
Administration of milk replacer to lambs	0.100	0.07
Application of reproductive control practices in the far	–0.017	0.76
Source of replacement animals	–0.030	0.60
Newborn care and specific monitoring	0.059	0.29
Maintenance of a colostrum bank	–0.045	0.42
Lamb fostering to female animals other than their dams	–0.024	0.67
Age of farmer	–0.140	0.012
Length of previous animal farming experience	–0.139	0.012
Highest general education level achieved	–0.033	0.55
Farmer by profession	0.123	0.027
Daily period of presence at the farm	0.035	0.53
Family tradition in farming	–0.020	0.72
Presence of working staff at the farm	0.107	0.06
Temperature at 2 m for the year preceding the visit	0.019	0.74
Temperature of Earth skin for the year preceding the visit	0.026	0.65
Minimum temperature at 2 m for the year preceding the visit	0.131	0.018
Maximum temperature at 2 m for the year preceding the visit	–0.085	0.13
Temperature range at 2 m for the year preceding the visit	–0.129	0.020
Relative humidity at 2 m for the year preceding the visit	0.098	0.08

Precipitation for the year preceding the visit	0.103	0.07
Wind speed at 10 m for the year preceding the visit	0.087	0.12

Table S4. Results of univariable analysis for predictors of incidence rate of respiratory problems in kids in 119 goat herds in Greece.

Variable	r_{sp}	p
Management system applied in farm	–0.038	0.68
Part of the country where farm was located	0.084	0.37
Altitude at the location of farm	0.230	0.012
Availability of a milking parlour	0.097	0.30
Availability of a separate barn for kids	–0.052	0.57
Availability of a separate kidding area	0.046	0.62
Proximity (< 10 km) to industrial sites	0.291	0.001
Number of adult goats on the farm	0.242	0.008
Breed of animals	–0.008	0.93
Month of the start of the kidding season	–0.084	0.36
Average number of kids born per doe during the preceding kidding season	–0.042	0.65
Average age of culling does	–0.137	0.14
Collaboration with a veterinarian	–0.037	0.69
Total visits made annually by veterinarians to the farm during the preceding season	0.084	0.36
Vaccination against bacterial respiratory infections applied in the farm	0.063	0.50
Age for kid removal from their dams	–0.097	0.29
Annual frequency of systemic disinfections in the farm	0.098	0.29
Administration of selenium to pregnant animals	0.002	0.99
Administration of selenium to newborn animals	–0.020	0.83
Routine prophylactic administration of antimicrobials to newborns	0.260	0.004
Administration of milk replacer to kids	–0.045	0.63
Application of reproductive control practices in the far	–0.047	0.61
Source of replacement animals	–0.025	0.78
Newborn care and specific monitoring	0.072	0.43
Maintenance of a colostrum bank	0.030	0.75
Kid fostering to female animals other than their dams	–0.052	0.58
Age of farmer	–0.127	0.17
Length of previous animal farming experience	–0.053	0.57
Highest general education level achieved	–0.035	0.71
Farmer by profession	–0.078	0.40
Daily period of presence at the farm	0.074	0.42
Family tradition in farming	0.051	0.58
Presence of working staff at the farm	0.001	0.99
Temperature at 2 m for the year preceding the visit	0.027	0.77
Temperature of Earth skin for the year preceding the visit	0.029	0.75
Minimum temperature at 2 m for the year preceding the visit	0.131	0.16
Maximum temperature at 2 m for the year preceding the visit	–0.256	0.005
Temperature range at 2 m for the year preceding the visit	–0.143	0.12
Relative humidity at 2 m for the year preceding the visit	0.202	0.028

Precipitation for the year preceding the visit	0.026	0.78
Wind speed at 10 m for the year preceding the visit	0.114	0.22

Table S5. Complete results of analysis of incidence rate of respiratory problems in lambs and kids in 444 farms in Greece, in accord only with management system and proximity to industrial sites.

Variables	Relative Risk (± s.e. [1])	*p*
Sheep Farms		
Proximity of the farm to industrial sites		0.011
Yes (0.0% (2.2%))	1.013 ± 1.005	0.008
No (0.0% (0.5%))	reference	-
Management system applied in farms		0.28
Intensive or semi-intensive management (0.0% (0.8%))	1.005 ± 1.004	0.21
Semi-extensive or extensive (0.0% (0.6%))	reference	-
Goat Farms		
Proximity of the farm to industrial sites		< 0.0001
Yes (0.3% (6.7%))	1.040 ± 1.009	< 0.0001
No (0.0% (0.0%))	reference	-
Management system applied in farms		0.21
Intensive or semi-intensive management (0.0% (0.0%))	1.005 ± 1.004	0.21
Semi-extensive or extensive (0.0% (0.0%))	reference	-

Figure S1. Bi plot of results of principal component analysis for the parameters into the final multivariable assessments for incidence rate of lamb respiratory problems in sheep flocks with (black) or without (green) proximity to industrial sites (standard scaling, with no rotation during preprocessing).

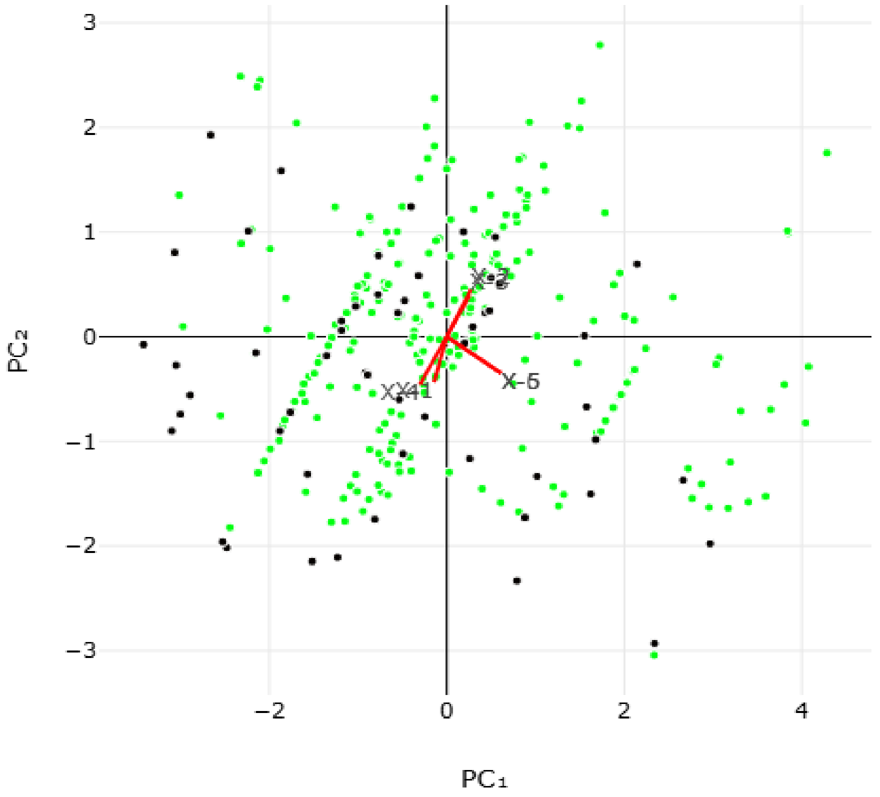

Figure S2. Bi plot of results of principal component analysis for the parameters into the final multivariable assessments for incidence rate of kid respiratory problems in goat herds with (black) or without (green) proximity to industrial sites (standard scaling, with no rotation during preprocessing).

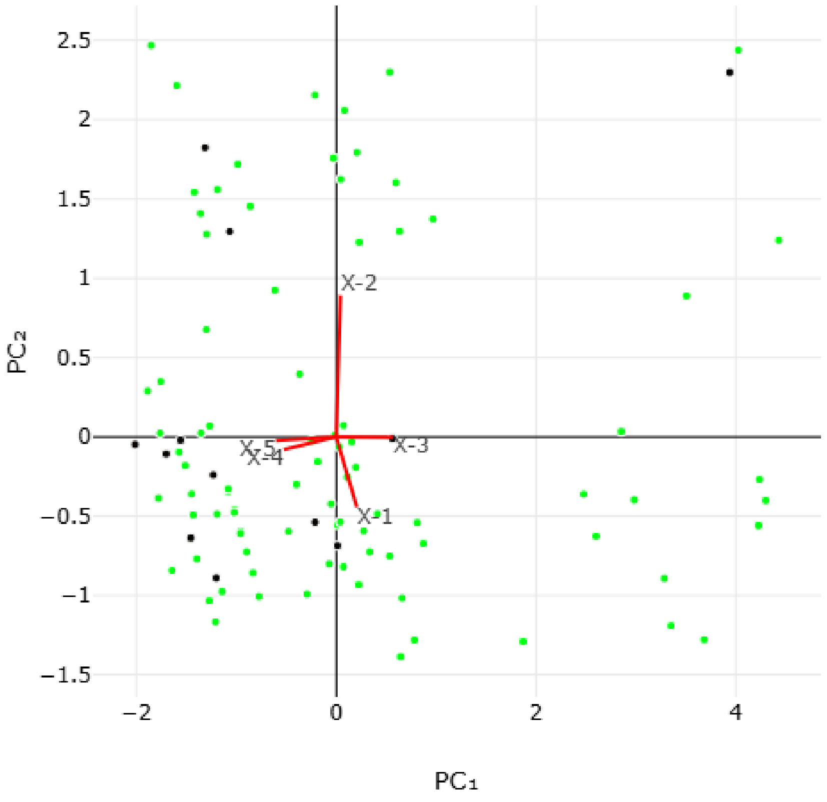

Supplement: Supplementary file 1 [file animals-15-03155-s001.zip › animals-3901460-supplementary.pdf]
